# P-1058. Characteristics and Real-world Outcomes of Patients Administered with Fecal Microbiota, live-jslm for the Prevention of Recurrent *Clostridioides difficile* Infection (rCDI)

**DOI:** 10.1093/ofid/ofae631.1247

**Published:** 2025-01-29

**Authors:** Sahil Khanna, Sanghyuk Seo, Min Yang, Viviana García-Horton, Yipeng Gao, Hannah Kim, Loren Ormenaj, Amy Guo

**Affiliations:** Mayo Clinic, Rochester, MN; Ferring Pharmaceuticals, Inc., Parsippany, New Jersey; Analysis Group, Inc., Boston, Massachusetts; Analysis Group, New York City, New York; Analysis Group, Inc., Boston, Massachusetts; Analysis Group, Inc., Boston, Massachusetts; Analysis Group, Inc., Boston, Massachusetts; Ferring Pharmaceuticals, parsippany, New Jersey

## Abstract

**Background:**

Recurrent *Clostridioides difficile* infection (rCDI) is common, with a prior CDI linked to a risk of up to 65% for subsequent recurrences. Fecal microbiota, live-jslm (RBL) is the first microbiota-based product approved in the US for the prevention of rCDI in adult patients following antibiotic treatment for rCDI. This study reports the characteristics and outcomes of patients with rCDI who were administered RBL in a real-world setting.

**Figure:**
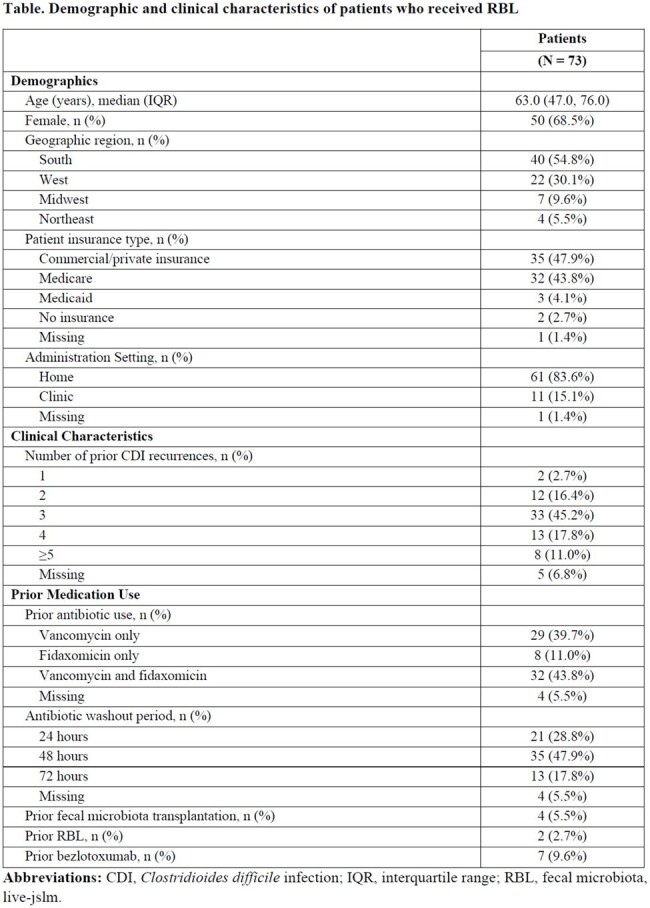

**Methods:**

Adult patients with rCDI who received RBL either at home or a clinic from healthcare professionals affiliated with a home-care provider network were included. Patients were required to have at least 8 weeks of follow-up after RBL administration, with CDI recurrence status assessed at week 8. Patient demographics, clinical characteristics, administration setting, prior medication use, and prior healthcare resource utilization were collected. The primary outcome was CDI recurrence within 8 weeks post-RBL administration. All variables were summarized descriptively using median and interquartile range (IQR) for continuous outcomes and count and percentage for categorical variables.

**Results:**

Between July 2023 and March 2024, 136 patients with rCDI received RBL, of whom 73 had at least 8-week follow up and reported CDI recurrence status at week 8. Patients had a median age of 63.0 years (IQR = 47.0, 76.0), 68.5% were female, most (74.0%) had ≥ 3 prior CDI recurrences, 5.5% had received fecal microbiota transplant, and 9.6% had received bezlotoxumab (**Table**). One third of patients (32.9%) had previously been hospitalized and 38.4% had prior emergency room visits due to CDI. Most patients were previously treated with antibiotics for rCDI: 39.7% with vancomycin only, 11.0% with fidaxomicin only, and 43.8% with both vancomycin and fidaxomicin. All patients, except for 4 with missing data, received RBL 24-72 hours post-antibiotics. Most patients (83.6%) received RBL at home. At week 8, the overall treatment success rate (no CDI recurrence) was 87.7% (87.3% [62/71] in RBL-naïve; 100% [2/2] in previously RBL-treated).

**Conclusion:**

RBL was effective in preventing recurrence of CDI at 8 weeks in a real-world setting, where most patients received RBL at home. These results suggest that RBL is effective in managing rCDI, including in a home care setting.

**Disclosures:**

**Sahil Khanna, MBBS, MS**, Ferring Pharmaceuticals, Inc.: Grant/Research Support|Finch: Grant/Research Support|Immuron: Advisor/Consultant|Niche: Advisor/Consultant|Pfizer: Grant/Research Support|Probio Tech, LLC: Advisor/Consultant|Seres Therapeutics: Grant/Research Support|Takeda: Advisor/Consultant|Vedanta: Grant/Research Support **Sanghyuk Seo, PharmD, MS**, Ferring Pharmaceuticals, Inc.: Employee **Min Yang, MD, PhD**, Analysis Group, Inc.: I am an employee of Analysis Group, Inc., which has received consulting fees from Ferring for the conduct of this study. **Viviana García-Horton, PhD**, Analysis Group, Inc.: I am an employee of Analysis Group, Inc., which has received consulting fees from Ferring for the conduct of this study. **Yipeng Gao, PhD**, Analysis Group, Inc.: I am an employee of Analysis Group, Inc., which has received consulting fees from Ferring for the conduct of this study. **Hannah Kim, PhD**, Analysis Group, Inc.: I am an employee of Analysis Group, Inc., which has received consulting fees from Ferring for the conduct of this study. **Loren Ormenaj, n/a**, Analysis Group, Inc.: I am an employee of Analysis Group, Inc., which has receivedconsulting fees from Ferring for the conduct of this study. **Amy Guo, PhD**, Ferring Pharmaceuticals, Inc.: Employee

